# Eye on Music Reading: A Methodological Review of Studies from 1994 to 2017

**DOI:** 10.16910/jemr.11.2.2

**Published:** 2018-05-01

**Authors:** Marjaana Puurtinen

**Affiliations:** University of Turku, Finland; www.utu.fi

**Keywords:** Expertise, eye movement, eye tracking, method, music notation, music performance, music reading, review, sight reading

## Abstract

In this review, we focus on the methodological aspects of eye-tracking research in the domain of music, published and/or available between 1994 and 2017, and we identify potentially fruitful next steps to increase coherence and systematicity within this emerging field. We review and discuss choices of musical stimuli, the conditions under which these were performed (i.e. control of performance tempo and music-reading protocols), performer’s level of musical expertise, and handling of performance errors and eye-movement data. We propose that despite a lack of methodological coherence in research to date, careful reflection on earlier methodological choices can help in formulating future research questions and in positioning new work. These steps would represent progress towards a cumulative research tradition, where joint understanding is built by systematic and consistent use of stimuli, research settings and methods of analysis.

## Introduction

Eye movement research has attracted increasing interest in recent decades as a fruitful approach to studying cognitive factors underlying domain expertise, including eye movements during music reading. Indeed, this approach to visual expertise research is well suited to the act of music reading, for a number of reasons. First, musical symbols can roughly be said to have motor counterparts. This enables the researcher to verify that each to-be-read symbol is being processed throughout the course of eye-movement recording, at least to the extent that it is correctly performed, as in studies of typing or reading aloud. This detailed, ongoing verification of the reading process is not achievable in many other natural visual tasks, such as silent reading of text or viewing of complex images. Second, as fluent music-reading and instrumental skills are not typically taught in general schooling but music is a profession for some, it is possible to identify performers with different levels of domain expertise, from novice to expert. Third, as a universally used system, Western music notation is not restricted by language borders. In addition, the impact of this work extends beyond academia: music-reading skill is relevant for both professionals and amateurs (and, indeed, for their teachers). As such, the research is of wide potential interest and has clear practical implications.

When reading music, our eyes do not move linearly across the musical score. Instead, and as with all visual processing, the reading consists of short moments when our eyes are somewhat still, called fixations, and rapid shifts between these ‘stops’, called saccades. In practice, during one fixation, we only see a few note symbols accurately and everything else in our visual array remains blurred. With a saccade we then move our area of accurate vision to fixate on the next note symbols, and to see them clearly. (For more information on eye movements, see [1, 2].)

It is now generally acknowledged that we only gain visual information during fixations and suppress it during the very fast saccades [2]. Thus, research of cognitive factors involved in visual tasks often studies the duration, location, order, and timing of fixations. In their review in 2008, Madell and Hébert set the average fixation duration during music reading at 200-400 ms [3]. More recently, however, Penttinen, Huovinen and Ylitalo [4] and Arthur, Khuu and Blom [5] reported slightly higher average durations (500-700 ms), and, overall, it seems likely that fixation durations are greatly affected by task- and performer-related factors. There is also large variability in the case of a single performer and a performance: Goolsby [6], for instance, noted that the fixation durations of one singer varied from 99 to 1640 milliseconds during one single performance.

To roughly summarize recent published work on music reading, eye movements are affected both by ‘top-down’ and ‘bottom-up’ factors (e.g., [4, 5, 7, 8, 9, 10, 11]). Not surprisingly, then, reading depends in practice both on ‘who is reading’ and ‘what is being read’. Regarding the former issue, increased expertise seems to have the overall effect of reducing average fixation durations and increasing fixation frequency during music reading [3, 4] (but see also Performance Conditions section below). This finding aligns with the reported actions of experts in some other domains [12]. Expertise may also result in an increase in how much the eyes are ahead of the ongoing performance [3, 11]. This distance, called the eye-hand span, has been suggested to average roughly around 1 s [4, 13].

However, less can be said about the issue of ‘what is being read’. One obvious drawback of previous work on music reading is that a focus on the effects of general expertise has directed attention away from the effects of the musical stimuli; indeed, in their review, Madell and Hébert [3] called for more work on the eye-movement effects of stimulus features. We can say that groups of note symbols seem to be processed, at least on occasion, as visual chunks (e.g. [7, 14]) and that violating melodic or harmonic expectations (by asking a musician to perform something that feels ‘wrong’ according to musical convention) causes performers to adjust their reading at the level of eye movement [4, 8, 15].

Overall, and even despite the early start by Jacobsen [16] and Weaver [17], this line of research is still at an early stage. It is therefore understandable that the field lacks methodological coherence and has yet to establish any standard approach. This absence of systematic research settings in this narrow field of study unfortunately hampers comparison and generalization of the scattered findings at any level of detail. In a growing area of research, with much to do and little to build on, we argue that a more detailed review of methodological choices in previous studies would be of benefit to researchers in formulating research questions and positioning new work, all in the interest of establishing a more systematic research tradition.

## Aim

The aim of this review is to support the crafting of more well-founded research hypotheses and the more systematic design of experiments in future work on music reading. To this end, we will review, in some detail, methodological choices in eye-tracking studies of music reading from 1994 to the present day, focusing on (a) choice of performed music, (b) performance conditions, (c) performers’ musical expertise, and (d) handling of performance and eye-movement data. In each section, we discuss how these choices may have affected interpretation of the studies’ findings and alignment, and we offer recommendations for increasing the field’s coherence. We focus on studies of ‘sight-reading’ during a musical performance (i.e. reading at first sight) (see also Performance Conditions section below) or reading with varying amounts of prior exposure to the performed material. We ignore non-performance music-reading tasks (sometimes called ‘silent reading’; see, [18]). In addition to lacking motor components, silent-reading tasks make quite different cognitive demands on the reader (e.g. note or chord identification, error detection), compared to reading music while performing.

## Selection of reviewed papers

The papers selected for this review had to fulfill a number of criteria. First, they had to be published in 1994 or after, and available in 2017. Year 1994 marked the slow but evident growth of interest in this topic, following publication of Goolsby’s two seminal papers in *Music Perception: An Interdisciplinary Journal*. Second, the papers had to be published in peer-reviewed journals and written in English. Third, papers had to include a task involving music reading and simultaneous musical performance, (i.e. singing, tapping rhythms, or playing an instrument). Through search engines and author contact, 15 publications were identified that met these criteria (Table 1).

**Table 1.** Selected papers: peer-reviewed scientific journal articles on eye movements during musical performance published in the English language since 1994

Author(s)	Year	Journal	Title
Goolsby	1994(a)	*Music Perception: An Interdisciplinary Journal*	Eye movement in music reading: Effects of reading ability, notational complexity, and encounters
Goolsby	1994(b)	*Music Perception: An interdisciplinary Journal*	Profiles of processing: Eye movements during sightreading
Kinsler & Carpenter	1995	*Vision Research*	Saccadic eye movements while reading music
Truitt, Clifton, Pollatsek & Rayner	1997	*Visual Cognition*	The perceptual span and the eye-hand span in sight-reading music
Furneaux & Land	1999	*Proceedings of the Royal Society of London*	The effects of skill on the eye-hand span during musical sight-reading
Gilman & Underwood	2003	*Visual Cognition*	Restricting the field of view to investigate the perceptual span of pianists
Wurtz, Müeri & Wiesendanger	2009	*Experimental Brain Research*	Sight-reading of violinists: Eye movements anticipate the musical flow
Penttinen & Huovinen	2011	*Journal of Research in Music Education*	The early development of sight-reading skills in adulthood: A study of eye movements
Ahken, Comeau, Hébert & Balasubramaniam	2012	*Psychomusicology: Music, Mind & Brain*	Eye movement patterns during the processing of musical and linguistic syntactic incongruities
Drai-Zerbib, Baccino & Bigand	2012	*Psychology of Music*	Sight-reading expertise: Cross-modality integration investigated using eye tracking
Penttinen, Huovinen & Ylitalo	2015	*International journal of Music Education: Research*	Reading ahead: Adult music students’ eye movements in temporally controlled performances of a children’s song
Rosemann, Altenmüller & Fahle	2016	*Psychology of Music*	The art of sight-reading: Influence of practice, playing tempo, complexity and cognitive skills on the eye-hand span in pianists
Arthur, Kuhn & Blum	2016	*Journal of Eye Movement Research*	Music sight-reading expertise, visually disrupted score and eye movements
Hadley, Sturt, Eerola & Pickering	2018	*The Quarterly Journal of Experimental Psychologie*	Incremental comprehension of pitch relationships in written music: Evidence from eye movements
Huovinen, Ylitalo & Puurtinen	2018	*Journal of Eye Movement Research*	Early attraction in temporally controlled sight reading of music

Note. One can observe a shift from more method-specific psychology journals towards domain-specific journals focusing on cognitive musicology and music education. This has most likely played a role in how authors have reported methodological aspects of their research, in turn influencing the issues discussed in this review.

## Performed Music

**Figure 1.**
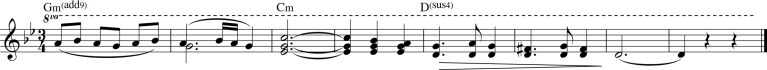
Example of one-staff notation (Source: Author MP).

To begin, we focus on the first issue mentioned above:‘what is being read’. Music notation can provide the performer with a wealth of information on the music in question; typically, the central elements are rhythm, melody, and harmony, along with other additional information (see 8-bar excerpt in Figure 1). Rhythms—that is, the lengths of individual notes and the patterns of their durational relationships—are implied by the stems, flags and heads of individual note symbols, which are then positioned between the vertical bar lines according to the given meter (see marking “3/4” in Figure 1). Rhythm relates to motor planning; in Figure 1, for instance, a pianist needs to make six successive key presses in bar one, whereas in bar three, only one chord— three simultaneously played notes—is performed. The melody—the succession of pitch heights—is reflected in the horizontal locations of concurrent note heads; in Figure 1, the melody first ascends slightly and then starts to descend after measures 3 and 4. Harmony is presented by groups of simultaneously performed notes or by chord symbols placed above the staff lines (see Figure 1), and additional information is given in textual form (e.g. instructions to perform the piece “vividly” or “slowly”), or by symbols. In Figure 1, the “8va” and dotted line signal that the whole sequence is actually performed one octave higher than where it is written, and the symbol below measures 5 and 6 indicate that the music should be played with decreasing loudness toward the end of the melody. Phrasing, signalled by note-binding arches as in Figure 1, has several meanings. A pianist regards the phrasing in measure 1 in Figure 1 as a guide for binding the notes as much as possible (more of an expressive guideline), whereas for a violinist the arch is also a signal for choosing the bowing for the measure, and for a clarinettist to use one single blow to perform it. In the final measure in Figure 1, the note-binding arch means that the last of the notes is not played, but the duration of the previous note is lengthened by the latter note’s duration.

In general, a performer tends to focus his or her gaze on note symbols or expressive markings that are relevant for motor execution, avoiding, for instance, vertical bar lines [6, 19, 20]. Fixations do not always land exactly on the note symbols, however; it seems to suffice to fixate close enough to a symbol to have it within in the area of accurate vision (see, e.g [19]). For the same reason, groups of notes may be inspected with single fixations [4, 6, 14]. In Figure 1, for example, it is likely that each of the three pairs of eighth-notes (joined with vertical beams) in bar one would be fixated on only once, as would the three-note chord in bar three. Importantly, written music only on occasion gives information about how to actually execute the note symbols. Instead, the motor protocol (which finger to use on a keyboard next, or which string and finger to use on the violin) needs to be either practiced beforehand or decided on the fly while performing.

Researchers have opted for one of the following two main approaches in terms of selecting performed music for their studies: the Natural Approach, where musicians are invited to perform authentic pieces, or the Experimental Approach, with specifically designed musical tasks. When applying the first of these (Table 2a), the focus of the studies has been in addressing global differences in eye movements during music reading with respect to the amount of visual information in the notated pieces [7, 21], performers’ skill levels and their perceptual and/or eye-hand spans [13, 20, 1013,] or the presence or absence of auditory models and/or fingerings [9]. To be sure, when studying expert-like music reading, the Natural Approach creates a more ecologically valid performing situation in which experts can use their domain knowledge and plan their motor responses to the stimuli exactly as they would ‘ordinarily’ do. This approach is very fitting for descriptive purposes—that is, when pointing out general pattern-like differences between reading by experts and novices, or when piloting and experimenting for future studies.

**Table 2a.** ‘Natural Approach’ studies and their musical stimuli (by year of publication)

Author(s)	Length	Stimulus description
*Two-staff system*		
Furneaux & Land	n.a.; ‘short’	Extracts from excerpts from piano pieces published under particular grade standards; different pieces for each skill level
Gilman & Underwood	3 bars	32 excerpts from piano chorales by J. S. Bach with tenor voice excluded
Drai-Zerbib et al.	4 bars	36 excerpts from tonal classical piano pieces
Rosemann et al.	30 bars	Excerpt from a piano accompaniment for flute sonata by J. S. Bach
*One-staff system*		
Goolsby (a and b)	n.a.; 4 staves	Four melodies from a collection of sight-singing exercises with some markings added by the researcher
Wurtz et al.	10 bars 21 bars	Two extracts from violin sonatas by Corelli and Telemann

**Table 2b.** ‘Experimental Approach’ studies and their musical stimuli (by year of publication)

Author(s)	Length	Stimulus description
*Two-staff system*		
Ahken et al.	5-7 bars	16 melodies composed for the study
*One-staff system*		
Kinsler & Carpenter	n.a.	Short rhythm tapping exercises; exact number n.a.; 32 trials in ‘a typical run’
Truitt et al.	9-18 bars	32 simple melodies from piano pieces by Bartok, slightly modified by the researchers
Penttinen & Huovinen	5 bars	12 simple quarter-note melodies composed for the study
Penttinen et al.	8 bars	A familiar children’s song and its two variations, composed for the study
Arthur et al.	4 bars	10 melodies composed for the study
Hadley et al.	8 bars	16 melodies composed for the studies
Huovinen et al.	5 bars (study 1)24 bars (study 2)	12 quarter-note melodies in study 1 and 8 quarter-note melodies in study 2, all composed for the studies

In the Experimental Approach (Table 2b), focus has been on the eye-movement effects of violating melodic and harmonic expectations [4, 8, 15], unusual visual layout [5, 8], or on the very basic reading mechanisms explored with extremely simple musical tasks [11, 14, 19, 22]. With simple tasks, the leading idea has been to keep some factors of the stimuli constant and only vary one: for instance, Kinsler and Carpenter [14] only asked their performers to tap rhythms, whereas Penttinen and Huovinen [22] and Huovinen et al. [11] created melodies where all notes were of the same duration (see also [19]).

In reviewing the findings of all these studies in parallel, the great variability in the stimuli and lack of consistency in creating them presents an obvious challenge; but this is especially so in the case of studies involving authentic music. As Figure 1 demonstrates, Western music notation is a complex symbolic system, where each note provides information about rhythm, melody, and harmony. These ‘chunks’ of information then form more or less conventional sequences and, in turn, still larger ‘chunks’ or patterns (at least for experts in this domain) (cf. [26]). For this reason, the lack of control over the visual information in the musical scores makes it impossible, in practice, to say what characteristics of the score may have caused the observed effects and why the experts or the novices read it as they did. How would we know whether those differences were an effect of musical expertise alone, or of the melodic or rhythmic elements of the music, or of a slightly less typical harmonic progression, or of difficulty in motor execution, or of a combination of some or all of these elements? We can only note differences; without a baseline understanding of the effects of various stimulus features in guiding eye movements, we cannot fully explain them.

Thus, the Natural Approach makes comparison across pieces challenging. In Goolsby’s [6, 21] studies, the one-staff stimuli contained not only note symbols but textual information and other types of markings referring to temporal and expressive features of the music. Similarly, in Wurtz et al.’s [7] study, violinists were given detailed information on bowing in one piece (signaled by note-binding arches) but not in the other (which, for the violin, means that each note is performed with its own bow movement). Comparing, for instance, average fixation durations across pieces with such differing amounts and types of information guides us at only a very general level. Another issue (as discussed later) is whether all performers actually focus on and/or execute all the instructions provided in the score.

The amount of information provided in the studies varies to the extent that, in some cases, pianists were required to read from two-staff systems, meaning that the music is written separately for the right and left hand (Tables 2a and 2b). As Weaver [17] noted in his early study, a two-staff system prompts vertical eye movements in skipping from one staff to the other (for illustrations, see [13]). Naturally, adding a staff often also adds to the visual information the performer must process and execute. Other studies employing one staff of music (as in Figure 1) eliminated the need to coordinate reading and performing from two parallel staves. These experiments studied either singers or violinists (who typically read only one-staff systems), or asked pianists to perform with only their right or left hand (Tables 2a and 2b).

As an example of the range of all this variation with respect to performed music and its visual layout, studies have investigated eye-hand span when performing a professional-level sonata accompaniment [10] or modified Bach chorales written for piano on two [20], one-staff tasks such as playing complex violin pieces [7], simple Bartok piano melodies performed with only the right or left hand [19], or a one-hand piano performance of a children’s song [4]. In addition, the length of music material varied in these studies from three to 30 bars, presenting the performers with very different conditions for the study of ‘looking ahead’. With short stimuli, the longest advance inspections (although very long ones seem somewhat rare) simply cannot occur. All this permits only broad overall comparisons between results, rather than a full meta-analysis of eye-hand span and the factors affecting it.

At the other extreme, analyses of eye-movement data based on the Experimental Approach (Table 2b) are of course, affected by their simplicity. Here, musicians do not need to perform at their maximum capacity. This relaxation of visual-motor challenges seems equally likely to affect the reading — especially for highly skilled performers (about selection of musical material for performers of different skill levels, see Performers’ Expertise Levels section below). However, following Madell and Hébert [3], we argue that to formulate hypotheses on expert-like behavior that go beyond the most general and advance this field of research, it will be necessary to devote greater attention to the systematic selection of stimuli when building research settings. Understanding the effects of the most basic features of music notation on the targeting and timing of eye movements seems essential before combining these observations with the effects of expertise, added visual elements, violation of musical expectations in complex settings, or even the distribution of attention between two staves.

To be sure, tasks designed according to the Experimental Approach can be quite far away from every-day music-making. It is therefore important to keep in mind that these simplified tasks are not the ‘actual’ targets of study: the findings we are after are not about the size of the eye-hand span in a certain task and for particular groups of performers, even though these may be the results of single experiments. Instead, we wish to move, one step at a time, toward understanding the process of transforming read note symbols into motor activity—and with musical meaning. One way to proceed is to systematically revisit previously studied stimuli under different conditions, or to modify or contrast them. This systematic commentary of tasks applied in prior research would aid in gradually moving toward the use of more complex musical stimuli. The work of Kinsler and Carpenter [14] on rhythm reading or of Penttinen and Huovinen [18] and Huovinen et al. [11] on reading of large melodic intervals (i.e. large “skips” between two consecutive pitch heights) may serve as useful points of departure for building an understanding of the effects of these music-structural features on eye movement. Their stimuli could quite easily be re-tested as well as complemented: melody could be added to the rhythms of Kinsler and Carpenter, and different rhythm patterns to the two other studies.

## Performance Conditions

Having decided on the appropriate stimuli in accordance with a set of research questions, two key issues to be considered with regard to performance conditions are: the time allowed for completing the task and whether performers should be allowed to familiarize themselves with the music before the performance.

### Control of performance tempo

In studies of visual-motor skills and domain expertise, music reading is unique by virtue of the temporal restrictions imposed on the reading task. In ‘correct’ performances, the reader must proceed within the given temporal framework and adjust his or her reading accordingly. Consider, for example, a pianist reading and performing the excerpt in Figure 1. During the performance, any increase in time spent on fixating on any of the musical symbols (e.g. working out the rhythmic pattern of bar 2) is time spent away from inspecting another (e.g. checking which keys to press for the chord in bar 3). If the performer stops at difficult sections, they violate the flow of the music, which is exactly what beginners or less skilled sight-readers tend to do [6, 24]. This is unlike text reading, where the reader can spend more time on difficult sections.

When reading music, each symbol has a specific relative duration as defined by the selected tempo. In most prior studies, however, performance tempo has not been controlled for, and participants have typically been allowed to choose their own. Consider, again, the example in Figure 1; if one performer chooses a relatively fast tempo and plays the excerpt in four seconds while another plays it in seven seconds, it is obvious that the latter performer simply has more time to fixate on the symbols. Given such differences in the total trial time, should we, for instance, compare average fixation durations? Furneaux and Land [13] as well as Rosemann et al. [10] reported in their studies (both with nine pianists) that, compared to a faster performance tempo, a slower tempo increased the time lag between fixating on a note and subsequently performing it. Thus, differences in tempo allow some performers more time to fixate upcoming music (see also [11]), making it difficult to compare eye-hand span and related measures across participants.

Reports that more skilled sight readers read with shorter fixation durations than poorer ones (see Introduction) are, in fact, based mainly on studies where more experienced performers also performed tasks faster than those with less experience [5, 19, 20]. For that reason, it is impossible to know how these skill-based groups may have differed at eye-movement level had the tempo been kept constant. The observation has been repeated under temporally controlled conditions only by Penttinen et al. [4], where two relatively experienced groups of musicians performed a children’s song. Only Penttinen and Huovinen [22], Penttinen et al. [4], Rosemann et al. [10], Hadley et al. [15], study 2, and Huovinen et al. [11] have reported keeping performances comparable in terms of tempo, and only the last of these studies systematically included tempo in the modelling process. Furneaux and Land [13] silenced their metronome after two beats, while Goolsby [6, 21] and Truitt et al. [19] gave the participants a tempo prior to the performance. However, in these latter studies, the reported performance durations indicate that the intended tempi were not maintained by all participants. In some studies, exact tempi were not reported, making them impossible to replicate.

In sum, this quest for a ‘natural’ approach also allows musicians to decide their tempo and so constrains the possibilities for eye-movement analyses. (In reality, as performers in orchestras, bands or singalongs often read and perform in a tempo selected by others, and many practice solo with a metronome, controlling the tempo is perhaps not as untypical as researchers have supposed.) By implication, the issue of ‘time’ should be carefully considered in this particular form of reading task and should be controlled for as needed to support proper testing of a research hypothesis. The use of a metronome or other means of maintaining temporal similarity across performances (e.g. playing with a recording; see [10]) makes it possible to study the allocation of fixation time across symbols, as well as looking ahead, without any blurring of effects by differing trial times. So far, only Huovinen et al. [11] have reported analyses of the interplay of set tempi and selected eye-movement variables that are based on a data set including several correct performances of simple melodies by more than just a few participants. Thus, there are also several research questions unanswered in relation to performance tempo alone.

### Sight-reading or rehearsed reading?

Most of the studies featured in this review focus on what has been called sight reading (see Table 1 for journal titles). Fluent sight reading is indeed a skill required by, for instance, professional orchestra musicians or accompanists. With huge repertoires, they rely heavily on their ability to perform notated music accurately and with appropriate interpretation after very little practice. However, definitions of sight-reading vary in the music literature and, as a consequence, in related research. For instance, Lehmann and Kopiez [25] characterized sight reading as ‘non- or under-rehearsed music reading [that] aims at an adequate performance in terms of tempo and expression’, and in many eye-tracking studies, performers have been allowed more or less prior exposure to the music in accordance with this definition. In fact, only Furneaux and Land [13], Penttinen and Huovinen [22], Ahken et al.[8], Rosemann et al.[10], Hadley et al.[15], and Huovinen et al. [11] have clearly stated that their sight-reading tasks were performed with no preview of the music. In some other cases, the same stimuli were used in different conditions [4, 5, 20], or reading while performing followed silently reading the music beforehand [9]. Only Truitt et al. [19], who allowed participants to practice half of the melodies, report statistical testing for preview effects. A number of studies [6, 10 13, 14 ,21 ,22] have deliberately investigated repeated performances of the same material.

Despite these differences in research protocols and whether the study focuses on eye movements during initial or later performance, all of these papers refer to their task as ‘sight-reading’ (for an exception, see [4]). However, when analyzing music reading at the eye-movement or cognitive level, it seems likely that the first encounter plays a role that differs significantly from later readings, where motor responses may have been planned either while silently studying the music or even during physical practice beforehand. Again, to enhance the coherence of this research, it would seem sensible to make more consistent (and explicit) use of the term ‘sight reading’, distinguishing that task from later encounters with the same musical material that might be characterized as ‘rehearsed reading’ [26]. Not surprisingly, repeated readings and increasing familiarity with the score seem to affect visual processing [6, 21]. However, this issue has been neglected and requires further exploration in settings that carefully select music stimuli and control performance tempo.

## Performers’ Expertise Levels

With regard to performers’ skill levels, our review indicates that three approaches have dominated earlier work; either one group of performers has been selected as representing (presumably skilled) performers or participants have been divided into groups, based on their musical background or, more specifically, on their sight-reading skill. In the first category, studies applying what we refer to as the Skilled-Only Approach (see Table 3a) have examined one group’s reading of authentic material [7, 10] or of more experimental performance tasks [4]. In practice, the focus has often been on the effects of certain stimulus characteristics, although the interpretation of the findings has been hindered by a lack of control of stimuli and study conditions.

**Table 3a.** Study participants and their musical background (those included in the final analyses in parentheses); ‘Skilled-Only Approach’ studies (by year of publication)

Author(s)	N	Reported level of expertise
Kinsler &Carpenter	4	‘Competent musicians’
Wurtz et al.	7	Violinists (23-76 years), ‘all trained’, four reportedly professionals
Ahken et al.	18	Pianists (17-45 years); average of 17 years of training
Rosemann et al.	9*	University students majoring in piano, skill level ‘assumed high’
Hadley et al.(Study 1)	30 (24)	Active pianists (18-66 years); all with ≥ 9 years of formal musical tuition; 20 of them for over 10 years
Hadleyet al.(Study 2)	33 (24)	Active pianists (18-69 years); all with ≥ 6 years of musical tuition
Huovinen et al. (Study 2)	26 (14)	‘Professional-level’ pianists (20-58 years) with ≥ 7 years of practice; average of 19 years of training

* For one set of statistical analyses, two groups of three pianists were compared.

Papers reporting the use of what we call the Sight-Reading Skill Approach (see Table 3b) focus on performers whose overall performance ability and musical background is assumed to match but who differ in terms of their sight-reading ability. In other words, these studies specifically study between-group differences but among trained musicians. (Again, however, the reader is reminded of the different definitions of ‘sight-reading’ in these studies; see previous section.) In the studies by Goolsby [6, 21] and Gilman and Underwood [20], participants were selected according to background criteria, and their sight-reading skills were pre-tested. The internal coherence of these groups supported the creation of hypotheses, ensuring that observed differences were due to effects of sight-reading skill rather than, for instance, performance (motor) abilities. In Gilman and Underwood [20], the highest grade level was used as a general reference point (see also [5]). Along with Goolsby [6], these studies illustrate the importance of separately assessing sight-reading and performance skills. Clearly, even among these high-level performers, there are still great differences in sight-reading skills. Unfortunately the failure to fully control tempo in these studies meant that better sight-readers were quicker in performing tasks. Having established this, the same sampling approach could be used in modified research settings.

**Table 3b.** Study participants and their musical background (those included in the final analyses in parentheses); ‘Sight-Reading Skill Approach’ studies (by year of publication)

Author(s)	N	Reported level of expertise	
Goolsby (a)	24	Graduate students of a school of music	
		Group 1:	12 with high scores on a singing achievement test
		Group 2:	12 with low scores on a singing achievement test
Goolsby (b)	2	One poor and one skilled sight-singer selected from Goolsby (a) above	
Gilman & Underwood(Task 1)	40 (30)	Pianists (8^th^ grade completed)	
		Group 1:	17 good sight-readers based on a sight-reading test
		Group 2:	13 poor sight-readers based on a sight-reading test
Gilman & Underwood(Task 2)	40 (14)	As in Study 1	
		Group 1:	9 good sight-readers based on a sight-reading test
		Group 2:	5 poor sight-readers based on a sight-reading test

The ‘Musical Background Approach’ represents the most typical way of addressing skill differences in empirical studies of expertise. Here, performers with differing levels of musical expertise were invited to participate (Table 3c). Musical background was typically established by means of background questionnaires, and some studies reported post hoc checks on performance duration or accuracy in experimental tasks [21, 22]. However, these studies varied considerably in approach, especially in their definition of ‘less-skilled’ performers, who ranged from complete musical novices to ‘novices’ with little training, and from ‘non-experts’ with some prior training to students minoring in music education (see Table 3c). As in the Skilled-Only Approach, it is therefore somewhat challenging to assess performance levels across participants in the different studies. For instance, the ‘non-experts’ in Drai-Zerbib et al. [9] and Arthur et al. [5] may share more similar backgrounds than the ‘active pianists’ who were sole representatives of music readers in Hadley et al. [15] (Table 3a). More standardized pre-performance and sight-reading tests would aid comparison of these findings, as would more systematic vocabulary for describing participants.

For all studies involving participants with differing performance or sight-reading abilities, the selection of musical stimuli is undoubtedly a significant issue. Furneaux and Land [13], for instance, resolved this issue by presenting participants with pieces that matched their skill level, but this meant that stimuli were completely different across the three skill-based groups. In other studies, less skilled performers and/or sight readers have been made to struggle through tasks that were too challenging for them. For example, in Goolsby’s [6] illustrative case study, it was apparent that the poorer sight singer (who could barely perform the tasks at all) was unable to process all the information while the skilled sight singer performed the melody and the expressive and temporal markings with greater accuracy. It seems likely, then, that with such differences in sight-singing skills and outputs, the material was not even used in the same manner by the two readers. Gilman and Underwood [20] also report significant data loss in terms of performance accuracy, especially in their study 2 (see Table 3b). It remains unclear whether the skill-based groups of prior studies that produced very different performance outcomes were performing the ‘same’ tasks; while some excelled in expressive interpretation, others struggled to get through.

To overcome these difficulties, some Musical Background Approach studies used stimuli that were simple enough to be performed correctly even by less-skilled performers (see [4, 22]), as did some studies applying the Skilled-Only Approach [11, 14, 15]. Here, the idea is to examine performances that are as similar as possible, minimizing performance errors. Naturally, again, the task is easier for some than for others, but at least the outputs are similar in terms of the performed music.

**Table 3c.** Numbers of study participants (with those included in the final analyses in parenthesis) and their musical background for the Musical Background Approach studies. Arranged by publication year.

Author(s)	N	Reported level of expertise	
Truitt et al.	8	Pianists with 2-16 years of experience; average of 10 years of piano experience and 7 years of formal musical tuition	
		Group 1:	4 with slower average performance time per bar
		Group 2:	4 with faster average performance time per bar
Furneaux & Land	8	Pianists
		Group 1:	3 novices (appr. Grade 3-4)
		Group 2:	3 intermediates (Grade 6-7)
		Group 3:	2 professional accompanists
Penttinen & Huovinen	49 (30)	BA (education) students (20-41 years)	
		Group 1:	15 novices with no music-reading skill or instrumental training
		Group 2:	15 amateurs with music-reading skill and ≥ 1 year(s) of instrumental training
Drai-Zerbib et al.	25	Pianists
		Group 1:	10 non-experts with 6-8 years of training
		Group 2:	15 experts with > 12 years of training**
Penttinen et al.	40 (38)	Music students (17-37 years)
		Group 1:	24 music education minors
		Group 2:	14 music performance majors
Arthur et al.	22*	Pianists (18-21 years)	
		Group 1:	13 non-experts not performing at 6th grade level
		Group 2:	9 experts performing at 6th grade level
Huovinen et al.(Study 1)	37	Music students (17-37 years)
		Group 1:	23 music education minors
		Group 2:	14 music performance majors

* Arthur et al. (5) reported the total number of participants as 22, but the method section reports 20 participants.

** Final group sizes in the correlation analyses were 8 and 13,respectively.

## Performance and Eye Movement Data

In reviewing earlier studies and planning for future work, two further issues seem important: the quality and handling of performance data and eye movement data.

### Handling of performance errors

When a musician is asked to perform, there is always a risk of errors, even with highly skilled performers. As mentioned above, Goolsby [6] described in detail the differences between the struggling and fluent sight-singer, though both were skilled professionals. Gilman and Underwood [20] decided to use data from only 14 of 40 highly skilled participants when analyzing their second and very challenging performance task, which included transposing a chorale into a key other than that on the score. In studies with novices, too [22], the researcher certainly needs to find ways of dealing with erroneous performances.

On making an error, a performer typically either stops at that point to correct the mistake—disrupting the flow of the music and taking ‘too much’ time for the erroneous section—or continues to play something despite the errors made before subsequently returning to the ‘correct’ music. In such cases, the set therefore turns out to be incommensurate with either performance duration or similarity of output, or both. Until now, however, the eye-movement effects of performance errors during music reading have only rarely been addressed [6, 9, 22], and for good reason; one can go beyond case-level analyses only when performers commit enough of the same kinds of performance errors and at the same exact locations—and this rarely happens naturally. A case approach could be, of course, a good starting point (as in [6]), as it could lead to hypotheses for further group-level testing. In order to address the issue quantitatively, one could try to induce errors deliberately, with, for instance, an experiment where some ‘target’ notes would be changed without warning during a sight-reading performance.

All in all, given several participants and a task of sufficient difficulty, it is safe to say that performance errors will suffice to affect the millisecond-level eye-movement analyses, and they are worth their own study (see [6, 9, 22]). Nevertheless, many previous studies have included erroneous performances in their analyses. Goolsby [21], Kinsler and Carpenter [14], Furneaux and Land [13], Wurtz et al. [7], Drai-Zerbib et al. [9], Ahken et al. [8], and Arthur et al. [5] do not report the amount, type or effect of errors (either at all or in enough detail), pooling all performances in their analyses. However, in Kinsler and Carpenter’s [14] study, where skilled performers tapped rhythms, it is reasonable to assume that very few mistakes occurred. On the other hand, Goolsby [6] deliberately sought to illustrate the considerable variability in performances in his case study, but included all performances in his group-level statistical analyses (1994a). In other tasks that most often followed the Natural Approach in terms of musical stimuli and were not overly simplified, it is more than likely that errors did occur; indeed, Drai-Zerbib et al. [9] even reported correlations between performance errors and fixation time, suggesting that the performances were not of the same kind.

In some studies, limits were set to ensure that data would be accepted for analysis, which meant that most erroneous data were excluded. For example, Gilman and Underwood [20] calculated wrong and added notes in each (short) performance and required a minimum of 70% performance accuracy in task 1 and 60% in task 2. Hadley et al. [15] identified pitch errors and excluded participants who made errors in 50% of the experimental trials; of the remainder, 22% of trials included pitch errors. Conversely, Rosemann et al. [10] handled their data by excluding data points where at least four of the nine performers made a mistake. In their follow-up study, Penttinen and Huovinen [22] focused specifically on increases in novices’ performance accuracy and parallel changes in eye-movement patterns. They analyzed relative fixation durations and performed additional analyses of temporally stable performances to control specifically for temporal variability between the performances of novices and more skilled amateurs. Three studies [19]; data until the first error included, and [4, 11] reported that only error-free data were analyzed.

In summary, data sets that include performances differing in both overall trial time and local handling of tempo (where a performer stops at a mistake and then continues in the original tempo) make it difficult to draw meaningful conclusions about many basic eye-movement measures. In addition, reading processes are not directly comparable where some participants execute all the score information and others execute only some, perhaps erroneously (as in [6]), and steps should be taken to evaluate the degree of difference. Again, consistent handling of performance errors (and detailed description of how this was done) would facilitate comparison of findings related to eye-movement measures across different kinds of stimuli and for participants with varying musical skills.

### Statistical analyses

As shown in Tables 3a–3c, most of these studies involved small sample sizes. However, with the exception of Kinsler and Carpenter [14], they still base their findings on statistical analyses, even though these relate to groups of less than five participants. Granted the difficulty of finding large numbers of skilled performers, there are three ways of addressing this problem. First, as Kinsler and Carpenter [14] did with their four participants, one can look to more descriptive presentation of the data that may ultimately lead to expertise-related research hypotheses that are better than the piecemeal statistical analyses associated with extremely small samples. A more descriptive take seems as valid as a statistical analysis, which cannot be viewed as strong evidence for or against a given hypothesis when the sample size is small. A second approach is to design an experiment where the same participants perform a high number of trials. This approach would, naturally, require the use of statistical methods that take into account the dependencies between these measurements (see below). Something along these lines (though without statistical analysis) was applied by Kinsler and Carpenter, in whose study the four musicians typically tapped 32 simple trials. Yet another option is to ensure that performance tasks are simple enough for intermediate or amateur-level musicians, who are easier to find in greater numbers than high-level professionals. This approach has been applied by, for example, Penttinen and Huovinen [22], Penttinen et al. [4], Hadley et al. [15], and Huovinen et al. [11], using simple stimuli that could be performed by non-professionals. Data acquired in this way can also provide a stronger basis for studies of high-level experts, where a few experts can later be compared with the larger data pool of non-professionals.

In general, then, small participant numbers and a lack of controlled study conditions mean that great caution is needed in drawing conclusions from statistical analyses. In some cases, for instance, group sizes are too small to enable a single analysis of interactions between all factors of interest. Researchers have therefore had to analyze several factors separately (e.g. [10, 20]), which can generate overly strong effects for factors that are actually mediated by others. Additionally, it seems that when reporting ANOVAs, one may sometimes also interpret (or highlight) the main effects of factors that are also included in the interactions, though this should be done with care [27]. This procedure, which is common in the reviewed papers, can assign too much significance to some factors or unduly simplify their role in the complex act of music reading. Huovinen et al. [11] fitted factors of interest influencing the performers’ ‘looking ahead’ into one model and found main effects of expertise and tempo and, importantly, significant interactions between their selected stimulus characteristics. Analytical procedures of this kind seem fruitful for future studies, enabling them to go beyond noting general differences between participants or across different stimuli. Overall, care and precision in interpreting statistical analyses would bring us closer to explaining the interplay of the various ‘top-down’ and ‘bottom-up’ effects observed during music reading. Furthermore, and especially in an emerging field such as this, reporting of null findings and unexplainable interactions can be as informative as significant main effects and clear-cut interactions. Along with detailed description of research methods, reporting of such results may help the next research team to avoid the same pitfalls.

In addition to this general approach to (statistical) data analysis, it also seems important to consider the most appropriate eye-movement measures and to ensure their consistent use. Hyönä, Lorch and Rinck [27] sought to align concepts and measures used in text-reading studies, such as first-pass fixation duration and total fixation duration. Penttinen and Huovinen [22, 29] were apparently the first to apply these measures as defined to music-reading studies. The ideas underpinning these concepts (for instance, differentiating first and second pass fixations to a target area) have also been taken up by others, but there is ongoing variation in how these measures are named and, more importantly, in how they are calculated. Differences of operationalization clearly make the interpretation and alignment of findings more difficult. By way of example, fixation durations (either first-pass or total fixation times) have been calculated for individual notes [22], for equal-sized beat areas comprising 1 or 2 note symbols [4], for half-bar sized areas [22] and for full bars [8, 9, 15]. Adding to this mélange, researchers have reported findings based on the means of first fixations to a target (e.g. [9]), the sum of these (e.g. [4]), and their duration relative to the individual’s total fixation duration [22]. Alternatively, average fixation durations have been calculated for performance of a whole piece of music, regardless of where fixations landed [5, 7, 21]. This averaging or summing of data points produces distributions that are closer to normal and so permit statistical analysis, offering a way of eliminating dependency between observations. However, pooling of fixation data or removal of information about fixation locations can provide only partial answers to questions about the effects of performer characteristics and yields very little information about how music-structural features affect the reading. (The fixation data is, of course, also dependent on the recording frequency of the applied eye-tracker, which varies from 50 Hz to 1000 Hz in the reported studies, as well as on the manufacturers’ algorithms for defining a fixation).

The measures used to study the ‘looking ahead’ during music reading, often called the eye-hand span, exhibit similar variability. According to what Holmqvist et al. [2] give as the formal definition of the eye-hand span, it should be the lag between the start of a fixation on a particular note symbol and the starting moment of the same note’s subsequent performance (see also [7, 10, 13]. In music-reading studies the eye-hand span has, however, been more frequently calculated as the difference between a performed note and the concurrently fixated note (that is typically ahead of the performed one). This distance has been given either in milliseconds, pixels, notes or beats [4, 7, 10, 13, 19, 20]. Recently, Huovinen et al. [11] suggested a measure that compares the first fixation on a note with the on-going metrical time: they titled it ‘the eye-time span’ in order to separate it from those measures that relate fixation information to a motor activity.

All in all, as Hyönä et al. [28] have long since suggested to text-reading researchers, music-reading studies should systematize their measures in terms of both naming and methods of calculation. At this early stage of research, this remains a relatively easy task. Increased consistency and the cumulative evidence so gained should facilitate shared understanding of how these measures relate to surface- or deeper-level processing of musical stimuli and motor planning. In addition, music-reading researchers should closely follow current development trends in statistical methods for analyzing eye-movement data. These analytical tools may offer solutions to research questions that cannot fully be answered at present. For instance, although still in development, the modeling approach of Huovinen et al. [11] seems already to have produced more detailed information on the music-reading process than separate investigations of specific factors, while also accounting for dependencies within data sets.

## General discussion

In this review, we have discussed the methodological aspects of recent eye-tracking research in the domain of music and noted potentially fruitful next steps to increase the field’s coherence and systematicity. In particular, the review focuses on choices of performed music, the conditions under which it is performed (e.g. controlled tempo and music-reading protocol), performers’ levels of musical expertise and, finally, the handling of performance errors and eye-movement data for analysis.

While important progress has undoubtedly been made in many respects, there remains a clear need to ask and answer research questions concerning the basic elements of a music-reading task before embarking on more complex research designs where potential effects are blurred by other as yet unidentified factors. In particular, the effects of performance tempo have only rarely been addressed in a controlled way [10, 11, 13], and information generally remains scarce on the effects of most of the basic elements of music notation, including rhythm, melody, harmony and the placement of music on two staves. The differing definitions of ‘sight-reading’ suggest a need for separate study of initial encounters, where music is performed without prior exposure, and rehearsed readings (see for example [6, 21]). Importantly, we should also distinguish these acts by name (for instance, ‘sight-reading’ and ‘rehearsed reading’) [26]. In relation to eye movements, musical expertise (the defining of which should be more consistent) and performance tempo may well be more intertwined with the musical stimuli than has been thought and research settings and analytical choices should be created so that such complexities can be addressed (see [11]). Finally, the role of motor planning, which seems likely in particular to affect the need to ‘look ahead’ while reading, is only hinted at in studies asking participants to perform something ‘odd’ or ‘surprising’ and has not yet been systematically investigated. The fact that symbols must be executed at a given tempo is what makes music reading so interesting as a visual-motor task.

With a slightly more complete sense of the role of such characteristics, we could begin to explore in more detail the relation of sight reading and rehearsed reading to silent reading of music notation and other types of visual ‘reading’ (such as text or code reading), and to bridge studies about visual expertise in music with work done elsewhere on the performer-related characteristics affecting the music-reading skill (e.g. [30, 31, 32]). With respect to eye movements and the learning of music-reading skill, there is almost nothing but open questions; some studies do address the repeated reading and thus the learning of particular musical material [6, 10, 13, 14, 21], but the variability between the studies and lack of control in their designs hinder the making of strong conclusions. In addition, there is almost a complete lack of studies about beginners, as only Penttinen and Huovinen [22] have reported a data set that focused on ‘true’ novices in training. We should also keep in mind that there is still plenty of scope for more lenient, descriptive takes on this topic, creating research settings accordingly. Qualitative information gained in this way (as for instance in Goolsby’s [6] case studies) would help in formulating research hypotheses that could later be tested by a stricter statistical approach. No one researcher can tackle all these issues; thus, the benefits of a systematic, collaborative, and multidisciplinary study seem numerous.

Given the recent increase in research interest, we now have the box for the music-reading puzzle, but as yet, it contains only a few pieces. At this early stage, we have a wonderful opportunity to work towards a more coherent paradigm, in which research teams employ similar eye-movement measures and methods of analysis to build systematically on stimuli tested by others. Ideally, technical choices (related, for instance, to eye trackers and algorithms for defining fixations) would also converge. In pursuing those goals, the minimum requirement for now is to carefully report the detail of applied research designs; although this review has focused on the most basic elements of experimental studies (stimuli, task, participants, and data analysis), such details were not always provided in the reviewed papers. Precise descriptions of method and openness about successes and failures of choices made seem essential if other research teams are to learn from and build on each other’s work.

### Ethics and Conflict of Interest

The author(s) declare(s) that the contents of the article are in agreement with the ethics described in http://biblio.unibe.ch/portale/elibrary/BOP/jemr/ethics.html and that there is no conflict of interest regarding the publication of this paper.

### Acknowledgements

This research was supported by grant 275929 from the Academy of Finland, and the Turku Institute for Advanced Studies. We wish to thank Elke B. Lange, the anonymous reviewers, Hans Gruber, Erkki Huovinen, Anna-Kaisa Ylitalo and Nina Loimusalo for their comments on the manuscript, Kalle Pihlainen and Lauren Fink for proofreading, and the authors of the reviewed works for their significant input in the field of eye movements in music reading.
